# Supplementary material for: Interleukin-26 Has Synergistic Catabolic Effects with Palmitate in Human Articular Chondrocytes via the TLR4-ERK1/2-c-Jun Signaling Pathway
Source: Cells. 2021 Sep 21;10(9):2500. doi: 10.3390/cells10092500 (PMC8471695; doi:10.3390/cells10092500)
Supplement: Supplementary file 1 [file cells-10-02500-s001.zip › cells-1347108-supplementary.pdf]

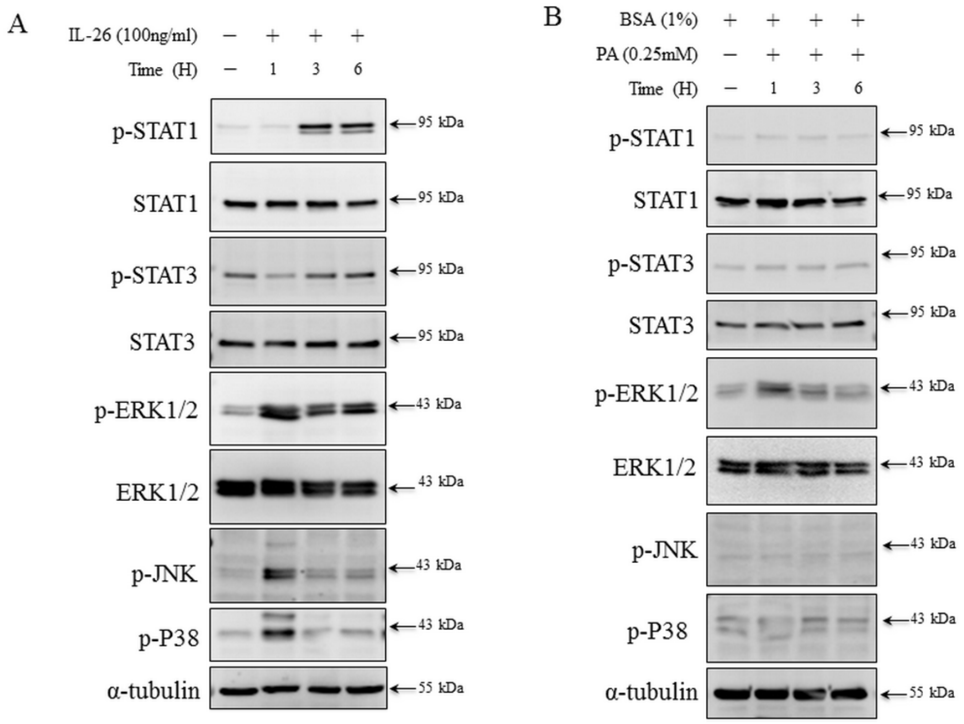

Supplemental Figure S1. IL-26 and palmitate induced activation of several signaling pathways in HACs. Cells were untreated or treated with 100 ng/ml of IL-26 and with 0.25 mM palmitate for 1 h under serum-free conditions. The cells were harvested and proteins were extracted for analysis of several signaling pathways by western blot analysis immediately after treatment and 1 h after depletion of (A) IL-26 and (B) palmitate.
